# Modes of Interaction in Naturally Occurring Medical Encounters With General Practitioners: The “One in a Million” Study

**DOI:** 10.1177/1049732321993790

**Published:** 2021-03-04

**Authors:** Olaug S. Lian, Sarah Nettleton, Åge Wifstad, Christopher Dowrick

**Affiliations:** 1 University of Tromsø–The Arctic University of Norway, Tromsø, Norway; 2 University of York, York, United Kingdom; 3 University of Liverpool, Liverpool, United Kingdom

**Keywords:** doctor–patient interaction, medical encounter, uncertainty, sociology, narrative analysis, England

## Abstract

In this article, we qualitatively explore the manner and style in which medical encounters between patients and general practitioners (GPs) are mutually conducted, as exhibited in situ in 10 consultations sourced from the *One in a Million: Primary Care Consultations Archive* in England. Our main objectives are to identify interactional modes, to develop a classification of these modes, and to uncover how modes emerge and shift both *within* and *between* consultations. Deploying an interactional perspective and a thematic and narrative analysis of consultation transcripts, we identified five distinctive interactional modes: question and answer (Q&A) mode, lecture mode, probabilistic mode, competition mode, and narrative mode. Most modes are GP-led. Mode shifts within consultations generally map on to the chronology of the medical encounter. Patient-led narrative modes are initiated by patients themselves, which demonstrates agency. Our classification of modes derives from complete naturally occurring consultations, covering a wide range of symptoms, and may have general applicability.

Based on a qualitative analysis of 10 naturally occurring primary care consultations in England, we explore the ways in which medical encounters are conducted in situ as a collaborative enterprise. By analyzing speech acts from complete verbatim transcripts, our exploration covers all consultation aspects, from diagnostic and etiological issues to treatment options and prognosis. Our core purpose is to draw out modes of interaction exhibited in the data. By “mode”, we mean a particular way of *doing* the interaction. When patients and general practitioners (GPs) interact, they *do* it in a particular manner and style, mainly conveyed through verbal actions. Interaction is a process, and it is the interactional dynamics that we seek to explore. Our main objectives are to study naturally occurring talk between GPs and patients to (a) capture the manner and style in which the medical encounters are mutually conducted, (b) generate a model of *modes of interaction* that may have general applicability, (c) explore how interactional modes vary *within* and *between* consultations, and (d) explore how mode shifts come about *within* each consultation, including who initiates them. To strengthen our arguments, our in-depth analysis of the 10 consultations is related to our complete data set, which contains 212 consultations.

We relate our questions to the broader issue of patient-centered care, particularly emphasizing agenda- setting, distribution of “dialogic space” (Bakhtin, 1981), power-balance, shared decision-making, and coconstruction of illness narratives. Acknowledging patients’ right to make choices and take action based on their values and beliefs has been an important international ambition for a long time (World Health Organization, 2015). This ideal is founded on “respect for the dignity of patients as persons and recognition of their problems within the context of their lifeworlds of being” (Mishler, 1984, p. 6). Putting patient empowerment into practice means enabling patients to play a more active role in health-care settings by allowing them to enter a reciprocal “negotiation process” (Aujoulat et al., 2007, p. 772) in which their legitimate authority claims are acknowledged.

Previous cross-disciplinary research indicates that patients and doctors claim power by similar means such as introducing discussion topics, asking questions, interrupting the other, enacting “symbolic ways of constituting social identity” (Ainsworth-Vaughn, 1995, p. 270), structuring the discourse, and controlling the flow of information (Fisher & Groce, 1985, 1990). However, they are not equally successful. Although the play of power-relations within interactions is shifting, there is little evidence to suggest that clinicians are “surrendering their professional authority” (Timmermans, 2020, p. 266; see also Pilnick & Dingwall, 2011). Several systematic patterns of asymmetry in doctor–patient interactions are observed: Doctors often dominate interactions in terms of verbal activity and agenda-setting (Robinson et al., 2016); patients get ample opportunities to tell their stories (olde Hartman et al., 2013); patients avoid contesting doctors’ authority and expertise (Chiu, 2011); and patients who disagree generally either comply or leave (Chiu, 2011). Doctors and patients sometimes evaluate medical consultations differently (Röttele et al., 2020; Stokes et al., 2006), maybe because of conflicting role expectations and perceived breaches of “rules of conduct” (Stokes et al., 2006, p. 611). Lack of congruence between doctor and patient perspectives is associated with dissatisfaction and poor clinical results (Kornelsen et al., 2016).

In this article, we seek to expand this body of research both theoretically and empirically by drawing on situated speech acts to develop a theoretical model of interactional modes. Recent research tends to move toward more limited empirical data (specific patient groups and/or consultation aspects), collected through interviews or surveys. Our empirical data give us a unique opportunity to explore how doctor–patient interaction is actually conducted in social situations where “action is carried out” (Jerolmack & Khan, 2014, p. 202), rather than theorized. By using complete naturally occurring consultations with a heterogeneous sample of patients, we attain a holistic outlook on all consultation aspects related to a variety of conditions. Our research team is interdisciplinary (sociology, medicine, and philosophy), but our main analytical approach is sociological. Most importantly, this means situating doctor–patient interactions within the wider sociocultural contexts, which invariably permeate medical consultations, and exploring the relationship between the practice and the social field in which it is embedded.

## Theoretical Perspective

When people in need of professional health-care enter a consultation room, they also enter a specific *position* in a well-established social system and become “patients”. The architecture of the building and the layout of the rooms structure their entrance. Once inside, they interact in a social field constituted by a set of interrelated social positions. Actors who hold these positions face institutionalized “structuring structures” (Bourdieu, 1989, p. 18) that promote and counteract certain practices. The two main positions—doctor and patient—are complementary and asymmetrical. By virtue of their location in the social structure, doctors have an institutionally based authority position. When entering their positions in this field, actors become responsive to the preset repertoire of culturally shared norms and values they are expected to act upon. These informal and taken-for-granted rules of conduct are “tacitly claimed by each party” (Strong, 1979, p. ix), and they create a “ceremonial” order. The interaction is played out between participants who know—in the words of Bourdieu—“the immanent rules of the game” (Bourdieu & Wacquant, 1992, p. 99). Because of the “social genesis” of these rules, actors can choose to honor, invert, or disregard them (Strong, 1979).

Within this perspective, doctor–patient interaction is seen as embedded within, positioned, and inseparable from social context. Interaction takes place against a background of a shared stock of knowledge and normative expectations (Freidson, 1961; Schutz, 1954/1967). Participation requires familiarity with cultural norms, values, and beliefs that delineate purposes and boundaries of the social field (Bourdieu, 1977). Chains of speech acts in a dialogue must be understood in relation to the discursive frame of this field: They are derived from it, and they serve to reinforce it. The frame defines the limit of what Butler (1997) refers to as “acceptable speech” (p. 356), and it demarcates the line between the domains of the “speakable” and the “unspeakable.” Knowing the discursive frame means knowing how to communicate (how to speak and how to interpret an utterance) within a particular social field.

Within a social field, negotiations occur on both a *positional* level and an *individual* level. On a positional level, interaction between doctors and patients is negotiation per se. Negotiation is “built into the interaction” (Freidson, 1970, p. 322). It is the two *positions* that are negotiated: “Given the viewpoints of two worlds, lay and professional, . . . interaction in treatment should be seen as a kind of negotiation” (Freidson, 1970, pp. 321–322). Positions are negotiated between representatives who may or may not express diverging views. This does not imply that individuals lack agency, or that they invariably have divergent goals, but that individual doctors and patients act in—and from—specific positions that they represent, there and then. This means that when doctors and patients meet, it is “a complex process of social negotiation in which each party attempts to navigate the structural constraints and imperatives that their contradictory locations give rise to” (Wainwright et al., 2015, p. 19).

On an individual level, negotiations are performed through a verbal exchange of speech acts between individuals who build on and respond to each other’s utterances. Successful negotiation, defined as mutual agreement on a definition of a situation and a common course of action, often requires a cooperative spirit, mutual respect, shared goals, and shared decision-making. It also requires that all participants provide each other with a “dialogic space” where they freely can speak their minds.

## Data and Method

We approach our research questions through a qualitative analysis of verbatim transcripts of 10 naturally occurring GP consultations, sourced from the “One in a Million” data archive (Table 1). We applied for and received all data related to 212 consultations, which were all those with reason(s) for contact stated as musculoskeletal, cardiovascular, psychological, digestive, endocrine/metabolic/nutritional, neurological, or general/unspecified. Of the accompanying data (Table 1), we only use patient records.

**Table 1.** “One in a Million” Data Archive (Barnes, 2017; Jepson et al., 2017).

Type of study	A prospective observational study containing an initial data set, collected for future research and teaching purposes, and archived at the data repository of the University of Bristol, UK (http://www.bristol.ac.uk/primaryhealthcare/researchthemes/one-in-a-million/the-dataset/).
Data material	327 film- or audio-recorded and verbatim transcribed naturally occurring GP consultations collected between 2014 and 2015 in 12 general practices in and around the City of Bristol. Consultations take place between adult patients (aged 18–96) consulting with 23 different GPs. A total of 300 patients (90%) consented to data access by “other researchers, subject to specific ethical approval”. The data set also includes patient records (from current and subsequent related consultations, collected three months after the consultation), longitudinal patient pre- and postconsultation survey data (self-administered immediately before and after the consultation), sociodemographic data of patients and GPs, and GP practice data.
Funding	The National Institute for Health Research (NIHR) School for Primary Care Research (208) and the South West GP Trust.
Ethics	Ethically approved by South West–Central Bristol Research Ethics Committee (REC reference: 14/SW/0112).

*Note.* GP = general practitioner.

### Data Material

For this study, we selected a heterogeneous subsample of 10 of the 212 received consultations for in-depth analysis, using a maximum variation strategy based on patients’ gender, age, and reasons for contact. Among patients, we have seven women and three men aged 26 to 91 presenting with a wide range of symptoms (Supplemental Table S2). Their education levels range from secondary education to age 16, to higher university degrees. Two of them classify themselves as non-British (African and Asian). Among GPs, we have five women and two men aged 32 to 58, belonging to five different clinics.

### Data Analysis

Before we started the analysis, all four researchers simultaneously exchanged individually written notes on their own readings of all 10 consultations. Our notes were later compared and discussed in meetings with all researchers present to identify and explore divergent and convergent readings. Based on these discussions, we then performed a systematic data-driven thematic coding of the transcripts following a constant comparative method, whereby each new instance of a theme was compared with previous instances to thoroughly elaborate the properties of each theme (Glaser & Strauss, 2017). Themes were generated by coding all utterances, case by case, in NVivo (version 12.4), and predominantly structured in relation to the kind of utterances they entailed (declarative, suggestive, acceptive, explanatory, inviting, requesting, and so on). Our themes are thereby grounded in our data. After continuously discussing and revising our interpretations and classifications in several rounds, we created a final codebook of 78 coherent themes, which we used to code all 212 consultations. Coding results from the whole data set were later compared with results from our smaller sample by looking for divergence and convergence.

While building on the thematic coding results, we performed an in-depth narrative analysis of our selected 10 cases. By narrative, we mean taking each story (in our case, the dialogue) as a whole (not fragment into discrete categories), placing it “in the context in which it has been generated and told” (Bury, 2001, p. 281), and exploring the ways in which it unfolds. During the analytical process, we explored *what* was uttered (content), by *whom*, and *how* it was uttered (form). This investigation included ways of talking, how questions were raised, choice of words, and agenda-setting. Throughout the whole process, we treated utterances as speech acts (verbal actions that accomplish something, whether intended or not).

Systematic and transparent data coding does not outweigh the loss of information that occurs by decomposing collectively negotiated dialogues. In dialogues, meanings emerge through reciprocal *exchanges* of speech acts that derive meaning from each other. Answers, for instance, make little sense without the utterance that called it forth. By breaking a chain of speech acts in a dialogue, the ongoing dynamics of the interactional flow is lost. To preserve the context, we present a blend of dialogues and individual utterances.

Working with observation data has certain limitations. Most importantly, it prevents us from asking participants to elaborate their utterances. Including only 10 cases also prevents us from exploring differences between subgroups. Possible biases in the data relate to recruitment of GPs, who self-selected to take part in the study, and that participants might have been influenced by being conscious about being filmed.

### Ethics

Our study has received the National Health Service (NHS) ethics approval (Research Ethics Committee reference: 18/WM/0008; Integrated Research Application System [IRAS] project ID: 232578 dated 22. January 2018) and Bristol Data Repository clearance from the Data Access Committee (DAC). Ethical and legal issues pertaining to the data collection have previously been approved (Table 1). In this study, we only include participants who in the original study gave informed written consent for their data to be accessed and reused by *other bone fide researchers*, subject to NHS REC and Bristol Data Repository approval. All written data were anonymized upon receipt, and there has been no direct contact with research participants. The main ethical issues therefore relate to data protection. The data set is stored on a password-protected site at the University of York, UK, accessible to two researchers (Nettleton and Lian). To ensure participant confidentiality, we have abided the stipulations of the Controlled Data Access Agreement and Data Protect Act.

## Results

We identified five distinctive (yet partly overlapping) interactional modes in our data: question and answer (Q&A) mode, lecture mode, probabilistic mode, competition mode, and narrative mode. Text containing information that could compromise anonymity is indicated by “text omitted.”

### The Q&A Mode

All consultations begin in with what we label as “Q&A–mode” with GP-led Q&A sessions, where GPs ask “what”, “where”, “when”, and “how” to get patients to concretize their symptoms. Questions are particularly significant utterances because they request a response; they often entail a nudge; they “claim the right to control over the topic” (Ainsworth-Vaughn, 1995, p. 282) and, therefore, serve as a “rough index” to power in medical encounters (Ainsworth-Vaughn, 1995). Initial interview sessions last for up to 9 minutes and contain up to 25 questions. Typical questions ask, “what kind” of symptoms, “how” they appear and develop, “when” and “how often” they occur, and “where” they are located. To patients with pain, for instance, GPs ask:

- Chronic pain, remind me again, NAME, where is your pain?
- Have you had this pain before?
- So how long is this been going on for?
- What sort of pain? Is it dull or aching, or sharp or stabbing?

Questions tend to be pithy, sometimes limited to one or two words such as “Sneezing?”, “Sleeping okay?”, “Any pain?”, “Any fevers?”, “Any diarrhea?” and “Any blood?”. Patients derive meaning from such incomplete sentences through shared knowledge (Schutz, 1954/1967). To closed questions, patients usually answer “yes” or “no”. To open questions, they also respond briefly, sometimes as short as two to five words: “Around here”, “My back is awful”, “About a week”, “Only a bit more pain”, and “Most of the day”. Sometimes, they add adjectives or adverbs such as “uncomfortable”, “painful”, “awful”, and “horrendous”. A consultation between a 70-year-old woman and a woman GP that starts with 21 symptom-related questions, mainly of a closed kind, is illustrative:

GP: So the pain, is it kind of like a cramping pain?

P: Yes.

GP: No stabbing pains?

P: No.

GP: Is it there all the time?

P: No, not all the time, but mostly, yes.

The patient waits until less than a minute before she leaves, and nearly 11 minutes in the consultation, before she asks about results of her recent blood tests. Her case is typical, in the sense that, patients rarely ask questions, or in other ways try to set the agenda, during Q&A sessions. Normatively, the rule patients follow is to answer GPs’ questions politely and briefly, which reflects their normative position in the social field.

Types of questions asked is noteworthy. In a study of doctor–patient discourses, Drass (1982) classifies four types of questions that resonate well with our data: first, closed questions requiring yes/no responses, such as (all examples from our 10 consultations) “Have you had this pain before?”; second, closed questions to elicit confirmation, for instance, “So it’s quite significant?”; third, forced choice questions, which give optional responses such as “What sort of pain? Is it dull or aching, or sharp or stabbing?”; fourth, open information requests, which encourage more expansive replies, such as “So when you’re at home how are you feeling?”. Open “information request” is the only type of question that *structurally* gives patients the opportunity to articulate their concerns from their own perspective (Drass, 1982).

In our sample, GPs mainly use closed questions (often with accompanying response options) and rarely “information request,” especially not in the initial phase. At later stages, some GPs ask open questions that give patients more dialogic space:

- When you’ve had it before, what’s helped to make it better?
- Anything that you can identify that’s triggered that?
- In your mind what do you think we should do next?

The Q&A mode is by far the most dominating feature of the consultations. By being those who ask, GPs become “first movers”. This undermines patient centeredness by restraining patients in terms of what to express, and how to express it, and it might silence them. Patients are thereby left with narrow dialogic space, which means they get scant opportunity to communicate their experiences, views, and concerns in their own words. Because this mode dominates the opening stage, it plays a pivotal role. Q&A sessions are conducted for GPs to gain knowledge of patient’s conditions, but through their questions, they directly or indirectly frame patients’ problems and worries in a medical perspective. Thereby, the field is set for the remaining consultation: It places the meeting in a specific social field (a biomedical system) and a specific social situation (a medical consultation), and it locates both actors in specific asymmetric social positions (doctor and patient), which again reinforces the power-distribution between them. Although it often goes without saying, this element should not be overlooked because of this fundamental function of constituting the consultations.

In going to see the doctor, patients might have thought of themselves in medical terms already, but not necessarily in a biomedical sense, especially not if their problems are of an existential nature and they have nowhere else to turn (which is often the case in modern secular societies). In any case, the language provided by the GPs (re)enforce a biomedical perspective. As the Icelandic word for medicalization—“sjúkdómsvæðing” (Jónsson & Jónsdóttir, 2004)—so nicely captures (sjúkdóm = illness and væðing = transmission): Through processes of medicalization, biomedical views are *transmitted* to experiential phenomena. Ziółkowska (2009) has described something similar in a psychiatric setting: Through their questions, psychiatrists’ direct patients “to gaze at themselves and their actual problems” from a medical perspective (p. 1621).

Coding results from all 212 consultations support our interpretations: When GPs want patients to concretize their symptoms, they usually ask closed questions (Table 2). Patients answer politely and rarely ask questions (Table 3). We do, however, have to be cautious to compare GP and patient questions because patients tend to ask for information more indirectly than GPs (by question-like utterances such as “I do not understand”) and we only coded direct “unambiguous” questions (Ainsworth-Vaughn, 1995, p. 287).

**Table 2.** Direct Questions Raised by GPs to Patients (*n* = 212).

GPs Ask Patients Direct Questions About Current Symptoms by	Total Number of Questions
*Closed* questions with suggested answers (confirm, disconfirm or “forced choice”)	1,040
*Closed* yes–no questions	508
*Open* questions	436

*Note.* GP = general practitioner.

**Table 3.** Direct Questions Raised by Patients to General Practitioners (*n* = 212).

Patients Ask GPs Direct Questions About	Total Number of Questions
What is wrong with them, or what the cause of their illness might be	50
What the best treatment would be	41

### The Lecture Mode

The lecture mode typically first emerges when GPs communicate results of physical examinations and technological tests to patients. One example is from a consultation performed between a 75-year-old woman and her “usual” GP, who is a woman:

GP: Your cholesterol, the total level, is 5.2, which isn’t particularly high. When you combine it with your age, your blood pressure and your general risks—so weight, ethnicity, where you live—your risk of heart attack and stroke, still comes out quite high. The machine has calculated it on a very clever programme and it’s telling me that it’s around 27%. So that’s not quite 1 in 3. So over the next 10 years, you’ve got, almost, a 1 in 3 risk of having a heart attack or stroke. Now, we’d like to reduce that risk and I think you probably would too. How are we going to reduce it? Controlling the blood pressure is the first thing, but a cholesterol tablet to drop the cholesterol a bit more would be the other.

P: I mean, me and husband, he’s under you and we do have a healthy diet because of all his bits and pieces.

GP: A healthy diet helps, but it will make, maybe, 10% difference to the cholesterol levels. I think we’re not entirely sure whether it’s the cholesterol level itself, or whether it’s something that the cholesterol tablets are doing that makes the difference. There’s no doubt that cholesterol tablets do reduce the risk of heart attacks and strokes.

P: Well, it’s worth a try.

GP: Mmm. Unfortunately, we don’t have a crystal ball. If we put a whole lot of people in a room, we’re not going to be able to tell which one of those people will have the heart attack or stroke. We have to treat all of them.

P: I’d best take out life insurance, then. (laughter)

Here, the patient receives something that resembles a lecture in public health by a GP who seems cognizant of the population-level targets for cholesterol. The GP places the patient in a metaphorical room with a group of people and tells her she has an almost one in three chance of having “a heart attack or stroke”. The GP claims that medication “no doubt” reduces this risk more than dietary actions, which the patient mentions. The GP also explains that individual effects are uncertain but omits mentioning medication side effects. Astutely, the patient jokes referencing “life insurance” and laughs. Is she sarcastic or serious? Her laughter is a cue. Patients might use humor to create a soft outlet for emotions they find difficult to reveal directly (Schöpf et al., 2017). For our patient, the laugh might be a polite way of expressing fear caused by the rather bleak prospects she is given. The consultation ends with the patient agreeing to suggested medication, and the patient record reads “she is happy to start statin”, but the record for a revisit 4 weeks later reads, “backache & feet swollen after starting statin so stopped it again”.

During lecture-mode sequences, not all GPs present factual information (although sometimes presented as such). For instance, of the eight patients who receive information about technological test results, only two are informed about *actual* results (a pattern also seen in the whole data set, where GPs provide information about actual test results only in 31 of 212 consultations). Typically, GPs present their *interpretation* of test results, not factual information. They do so by using words such as “good”, “fine”, “normal”, and “reassuring”, or equally imprecise words such as “(a bit) high” or “(a bit) low”. Some of these utterances have a hint of closure to them, especially if the result is “good”:

. . . your blood pressure is actually really good today, which is reassuring.

Gosh, your oxygen level is a bit low.

Sometimes, GPs present findings in an impersonal manner, without reference to the person:

The blood pressure is better, but it’s not as good as I’d like it to be.

Pulse is normal.

Lecture-mode sequences mainly consist of GP-led monologues. GPs rarely inform patients about uncertainties related to interpretations of test results, and they rarely invite patients to discuss these issues. Patients rarely ask questions, interrupt or in other ways claim the floor during these sequences. All patients explicitly agree to at least one treatment suggestion. Uttering oral consent, however, does not necessarily mean convinced, and we do not know what they actually do (as with the woman who stopped taking statins). Patients might trust the GPs to know best, or act as they think is expected of them (follow the rules of the game). They also might think they have no real choice in the matter:

GP: There’s no doubt that cholesterol tablets do reduce the risk of heart attacks and strokes. [text omitted]

P: I’ll take the tablets.

In lecture-mode sequences, which are most pronounced when GPs elucidate results of technological tests and physical examinations, and when they suggest medical treatments, both GPs and patients act as expected from their positions in the social field. GPs express their superior expert role and institutional authority by providing information that patients rarely question or discuss (Table 3). Again, GPs’ way of talking enforces a medicalized perspective on patients’ problems.

### The Probabilistic Mode

“Medicine is a science of uncertainty and an art of probability” (Bean & Bean, 1961, p. 138). This famous Osler quotation captures the probabilistic mode that runs through all 10 consultations. GPs and patients hardly ever explicitly express absolute *certainty*. Among the very rare cases is a patient who is certain that her medication “must have helped, there’s no doubt about that”, and a GP who claims that “There’s no doubt that cholesterol tablets do reduce the risk of heart attacks and strokes”. Expressions of *uncertainty*, however, are frequently observed (up to 50 utterances in one of our 10 consultations, 30 from the GP and 20 from the patient). Both GPs and patients mainly express uncertainty indirectly, and they talk in probabilistic terms in relation to all consultation aspects.

As for GPs, they usually express uncertainties indirectly by words such as probably, possible, perhaps, plausible, (un)likely, may, maybe, think, not entirely sure, just to make sure, sometimes, hope, and watch and wait:

We would watch and wait that and should hope that that will settle down.

It’ll probably take best part of a week.

I don’t think there’s anything worrying going on.

I think what’s happened is that this is kind of muscular.

I think you do need something for your chest, actually.

A consultation between a 46-year-old woman who seeks help for musculoskeletal problems and hearing difficulties and a woman GP (not “usual” GP) is illustrative:

GP: I think most of the pain that you’re getting here, is coming from the muscles that move the neck and the one that shrugs the shoulders. They’re also quite sore to touch and really bunched up and tight. The other symptoms, like the hearing, may just be related to a viral infection you have at the moment. [text omitted]

P: So what’s happening? Because I’ve done something wrong?

GP: Sometimes, just an awkward movement will pull a few of the muscle fibers and then you get a reaction around them and it goes into spasm.

P: Alright, and then it will affect ___[0:12:46]?

GP: Yes, because the muscles in the neck attach on the bone just behind the ear and, also, at the back of the neck, here.

The GP uses probabilistic terms and explains she “thinks” both her the pain and her the hearing problems are related to bunched up and tight muscles (because neck muscles are attached to the bone behind the ear) after first saying that her hearing problems “may” be related to a viral infection. The two competing theories about her hearing difficulties are not further discussed and remain unconcluded, which dialogues conducted in a probabilistic mode generally do. During the next two months, the patient has four revisits and several telephone consultations related to the same problems.

In another consultation, a woman aged 57 presents leg and back pain to her “not usual” GP, who is a man, with florid adjectives such as “excruciating”, “burning”, and “stabbed”. She is limping because of the pain, and she feels “exhausted”. She worries that her condition might be related to her previous “malignant melanoma”, but she also proposes a diagnosis of sciatica. The GP seems to play down her symptoms by words such as “a bit irritated” and “a bit of local tenderness”. He replies that “I don’t think there’s anything worrying going on” and that he “don’t think” her pain is related to her previous cancer, while mentioning bursitis as a possible diagnosis. As often seen in cases of uncertainty, the GP concludes with a wait-and-see strategy, which includes some kind of medication (in this case, painkillers) and further investigation (in this case, various blood tests).

In similar ways to GPs, patients express uncertainties through probabilistic talk by using words such as think, probably, possibly, perhaps, maybe, not sure, and doubt. Such expressions relate to wide range of issues, including symptom descriptions (“I think it’s got worse”), etiology (“I just think it’s a chest infection”), and treatment effects (“I don’t think they work”).

The dominating role of probabilistic talk is hardly surprising because as noted above, medicine is inherently uncertain, which adds to the fragility of negotiations. Doctors and patients have to navigate possibilities, and if doctors assert certainty, they expose themselves to the possibility of being wrong. Conceptualization of uncertainty is “a potential safeguard against diagnostic error” (Miao et al., 2020, p. 1295). That patients embrace this way of talking is less expected; it might be that they somehow align to the ways in which GPs talk.

Our interpretations are supported by coding results from all 212 consultations: Both patients and GPs often express uncertainty and rarely certainty (Table 4).

**Table 4.** Total Number of Uncertainty and Certainty Words Used by GPs and Patients (*n* = 212).

Markers of Uncertainty and Certainty	GPs	Patients
*Uncertainty* words: probably, perhaps, possible/possibly, might/may/maybe, (un)likely, doubt, think, not sure, not certain, not know	2,396	1,475
*Certainty* words: obviously, certainly, no doubt	188	189

*Note.* GP = general practitioner.

### The Competition Mode

Another interactional mode observed in our data emerges when patients meet competition while trying to gain control of the dialogic space and set the agenda (what to discuss, and how to discuss it). The competition comes from GPs who indirectly challenge patients’ utterances, or disregard them by introducing new topics, sometimes through unwarranted remarks that seem to come out of nowhere. This breaches the chain of utterances and generates dialogues with broken chains. In a consultation between a 70-year-old woman with stomach problems and breathing problems and a “not usual” woman GP, this is particularly pronounced. This mode emerged as soon as the consultation began, which is unusual:

GP: How are you?

P: Well, not too good. Again, pains around here. I don’t know if it’s my gallstone. It’s still near to my bowel. The piles are more or less gone, but it’s not so sore but it’s still irritable. [text omitted] and my breathing. Yes, I’m not too good.

GP: Okay, so which one did you want to talk about most? [text omitted]

P: My stomach [text omitted]

GP: What makes you think it’s your gallstones?

P: I don’t know.

GP: Just because you know you’ve had gallstones?

P: Yes.

The patient’s first word is “well”, which signifies that a complex answer is to be expected. Then she says, “not too good” (an understatement?); hints to gallstone; refers to ongoing problems (“again” and “still”), and reiterates “not too good”. There are lots of opportunities here for the GP to follow up these cues, for instance, by asking her to elaborate. Instead, she closes off by asking if she thinks it is gallstone “just” because she has had it before, and which problem she wants to “talk about most” (patients are to book double appointments if they want to discuss several issues, and this might be a single appointment). “My stomach” the patient replies, but the GP proceeds by asking several questions about her smoking habits and thereby shows more interest in her chest. Thirteen questions and answers later, the patient repeats her gallstone theory, upon which the GP replies, “the thing is what we’re trying to do is keep you to one main doctor” (which she says seven times, in different ways, during the 12-minute-long consultation). Every time the patient tries to revert the discussion from chest to bowel, she is rebuffed. When the patient a bit later repeats her walking difficulties, the GP immediately heads this one off too by—again—linking to her chest:

P: I’m not walking too well. I feel weak in the legs.

GP: Well, I’m not surprised because your chest isn’t sounding great, to be honest. Have you got enough inhalers at home?

The GP also sidesteps the information about the stomach pain first starting after the patient experienced an assault by a man who is still her lodger:

P: Ever since that man attacked me I’ve been having trouble with my bowel. [text omitted] I don’t like men any more. [text omitted] He’s alright at the moment, but it’s day by day.

GP: Okay. What I’m going to do is I’m going to give you something for your chest and then I’m going to book an appointment with you to see Dr Name, okay?

P: Yes.

For the patient, the competition over what to talk about is once again a losing game: The GP responds to her two testimonies—“I don’t like men anymore” and “it’s day by day”—by offering her something for her chest and booking her an appointment with her usual GP. The patient politely responds “Yes”, which she does 50 times altogether during the consultation (on average 4.5 times each minute). By consistently acting in a polite and obedient manner, agreeing to whatever the GP says, the patient demonstrates adherence to the tacit rules of the game. However, her patient record shows 25 subsequent entries related to the same problems during the next 3 months. This might indicate that the rules of the game played out in the consultation room do not necessarily apply to patients’ wider interactions with their GPs.

A different kind of competition—more related to distrust than agenda-setting—is visible in a consultation between a 39-year-old man who struggles with itchy eyes, which he interprets as a medication side effect, and his “usual” woman GP:

GP: You went up to the eye hospital and they said they didn’t think it was anything to do with the medication; it was just a coincidence.

P: No, because . . .

GP: That’s what they said to me.

P: It was like a day or so later it was gone and I was fine.

GP: Oh was it? Oh, bizarre.

P: So I think because I stopped the medication, I think-

GP: You’re still convinced it was the medication? Okay.

P: Because it started when I took them and when I stopped taking them it corrected itself.

GP: It’s been a bit of a long journey that you’ve been poorly hasn’t it?

When the patient tries to explain (“No, because . . .”), the GP interrupts him (which she does 9 times altogether during the consultation) and asks, “You’re still convinced . . .?” which could be a polite way of saying that he is wrong. When the patient is allowed to fulfill his “because . . .” sentence, the GP responds by politely changing the subject. This indicates a lack of interest—and perhaps even distrust—in the patient’s experiences and judgments. The consultation then moves to his breathing problems, which the patient presents as “horrendous”. In the patient’s record, the GP describes him as a “Builder exposed to dust and asbestos”. Still, the GP responds to his symptom descriptions by an implied culpability criticism and insinuates that he might be using his asthma inhalers incorrectly (“a lot of people use inhalers incorrectly”):

P: I swear to God, I did. I used them all the time.

GP: Yes, with asthma you don’t necessarily need to use them all the time, you just use them when you’re bad.

P: That’s what I’m saying; I was doing exactly what they told me.

The ways in which the patient tries to present himself as a “good” patient (follow instructions) who is able (know techniques) and morally decent (work) are seen in many other consultations.

In another consultation, a 57-year-old woman who meets her “not usual” GP, who is a man, the patient explains how she is trying to deal with her back pain and limping:

P: I used to go to a chiropractor [text omitted]. I’ve managed my problems with good exercise, I’ve learned good exercise, McKenzie technique, so.

GP: Okay, yes.

The GP ignores this expressed evidence of patient expertise by a brief “Okay, yes”. Instead of following up her statement, the GP comments on her faulty shoes (“You need some different footwear on”) and offers anti-inflammatories.

In another consultation, a 51-year-old woman is coming to have her blood pressure measured, after recently being sent to the hospital with a suspected stroke. The patient describes the emotional strain of this experience to her woman GP:

P: I just thought I was dying.

GP: Did you? What was the outcome of that? Let me have a little look. [text omitted] Just looking further down the letter there’s nothing further to do there. That’s good. That’s reassuring. Okay, I’m up to date now.

The “little look” means reading the discharge letter from the hospital to become “up to date” herself. The GP never returns to the patient’s comment that she thought she was dying.

The competition mode is present in half of the consultations, and particularly dominating in three of them. Usually, patients perform their role by acting politely and refrain from directly subverting GP utterances, in line with Strong’s (1979) bureaucratic format: The gentility of patients is paired with doctors who exert “control over the shape and content of medical consultations” (p. 212). GPs rarely explicitly contest patients’ utterances, usually they just refrain from aligning them. Sometimes they prevent patients from setting the agenda and telling their stories more directly by cutting them off through short comments, before moving on to other issues.

Coding results from all 212 consultations indicates that both GPs and patients hardly ever *explicitly* express skepticism toward each other’s views on diagnostic and etiological issues (Table 5). When patients voice explicit skepticism, it is related to treatment suggestions. This might be because decisions about medical treatments are action-related conclusions of many consultations, with obvious practical implications, and therefore particularly important to patients. However, as our in-depth exploration of the 10 cases show, patients *indirectly* express skepticism in relation to diagnostic and etiological issues, especially the woman with the gallstone theory and the man with the itchy eyes. By doing so, they avoid overt confrontation.

**Table 5.** Total Number of Explicit Expressions of Skepticism From GPs and Patients (*n* = 212).

Explicit Skepticism Expressed in Relation to Each Other’s Views on	GPs	Patients
Diagnosis	1	1
Etiological explanations	13	2
Suggestions of medical treatment	37	113

*Note.* GP = general practitioner.

### The Narrative Mode

In sequences performed in a narrative mode, patients are given a dialogic space that enables them to tell their stories without major interruptions. For most patients, the dialogic space is quite narrow, but two women are given space enough to tell detailed stories to attentive listening woman GPs. One patient is a 51-year-old woman who meets a younger “not usual” woman GP. The GP opens the consultation by redefining the agenda from blood pressure control, which the patient explicitly asks for, to antidepressant (“Let’s recheck the blood pressure and talk about Fluoxetine”). A rapid mode shift toward a patient-led narrative mode emerges after the patient starts talking about previous counseling experiences:

P: I had counselling before. [text omitted] It didn’t make me any better. [text omitted] I saw him for a long long time. For some reason he had it firmly fixed in his head my problem were because I was abused as a child, but I wasn’t. I don’t know how . . .

GP: How did that come about?

P: I don’t know how he got all that. I absolutely was not. [text omitted] Then I saw a woman, I don’t know what she was, apart from very odd. She told me to imagine myself as an orchestra, not my kind of thing I’m afraid. She might want to live like that but I’m not going to . . .

GP: It was probably her technique. [text omitted]

P: I’m not going to no group thing.

GP: You don’t have to, you can say to them, “Look I prefer one to one.”

In the second case, a 35-year-old woman presents a story about workplace bullying to her “usual” GP, who is a woman about the same age as the patient. Most of the consultation is conducted in a narrative mode, initiated by the patient:

P: I’m going through a bad time at work at the moment, and I feel that I’ve been bullied, and I feel really stressed. I don’t sleep properly, worrying; don’t want to go back. [text omitted]

GP: So when you’re at home how are you feeling?

P: Because I did stay for three days and then I went back because I thought, “Okay, I can do it.” Went back and just answering the phone I would cry and I would feel really . . . I feel like I’ve been pushed out, they don’t want me there. And when I’m at home I do worry, and normally I would go out and about if I’m off; or see friends and family and just do my housework, but I just sit down and just been sad, and not eating properly. [text omitted]

GP: I wonder if you feel you’ve got enough support with your family and friends or whether you want someone professional to talk to about how you’re coping with it and how you’re feeling?

P: I don’t know. What do you mean when you say professional? Counselling is that? I don’t know.

GP: It’s early days, so I think if you feel you’re largely coping, I wouldn’t push you down that route.

P: Yes okay.

GP: But equally, if you feel you need it then I’m happy to-

P: To do it, okay. Yes, because then—if it’s going to help, because I have got three kids, and really I don’t want to be like this, and I am trying to push myself, so when the kids come back from school I’ve got to be normal. But when they’re out, as soon as I drop them to school, it’s like I’m just sitting on the sofa just thinking, because it is a horrible situation.

By setting the agenda, leading the dialogue and telling biographically anchored stories in a direct and apparently unreserved manner, these two women patients follow a different “script” than the others. An apt concept here is “patient orchestration”. Their utterances are nurtured by encouraging GPs who only interrupt their storytelling by asking for elaborations (“How did that come about?” and “when you’re at home how are you feeling?”) or offering short comments, never contesting views. By handing over the agenda-setting to patients, either silently or by invitation, these GPs provide the women with the dialogic space they need to communicate meaningful narratives. Patients’ storytelling is important because without it, “the possibility of diagnostic and therapeutic error increases, the likelihood of personal connections resulting from a shared experience diminishes, empathic opportunities are missed, and patients may not feel understood or cared for” (Connelly, 2005, p. 85).

Coding results from all 212 consultations is not easily compared with these findings, but there are some patient utterances that might indicate a narrative mode. Most importantly, it is common for patients to talk about issues related to work, family life, social networks, and illness management, and to express worries (Table 6). Occasionally, they also suggest how to interpret and medically manage their conditions (Table 6). The amount of such utterances varies a lot between the 212 consultations (from two to 72), but one in three (70 consultations) entail between 20 and 72 utterances of those listed in Table 6. This might indicate that the narrative mode is more present in the whole data set than in our smaller sample.

**Table 6.** Patient Utterances (*n* = 212).

Patients	Total Number of Utterances
– Describe social issues related to work, family life, and social networks	911
– Describe actions taken to manage their illness, including lifestyle	2,341
– Express worries	160
– Suggest interpretations of their condition, including causal explanations	121
– Suggest medical treatment	121

## Discussion

Patients and GPs create a choir of 20 unique voices, generating a polyphonic story. When performed together, each distinctive independent voice contributes to a consistent whole that can be described in numerous ways. We choose to describe it through their interactional dynamics, or mode dimensions.

### Five Interactional Modes

Common features exhibited in our data can be described through five interactional modes: Q&A mode, lecture mode, probabilistic mode, competition mode, and narrative mode (Table 7). The classification is based on the types of speech acts the dialogues entail. This is contrary to existing categorizations of doctor–patient interactions, which usually are based on normative categories, often with “paternalistic” included, for instance “paternalistic”, “informative”, “interpretive”, and “deliberative” (Emanuel & Emanuel, 1992), as well as the more recent classification of “controlled”, “constrained”, and “flexible” (Franklin et al., 2019). In addition, the relational dimension is missing in many previous classifications. Through our speech-act approach we stay as close as possible to the verbal actions that constitute the consultations, and by emphasizing mutual interaction more than individual contributions (such as “paternalistic”, for instance, which generally points to doctors only), we acknowledge the equally legitimate authority claims held by both parties.

**Table 7.** Modes of Interaction.

Mode	Main Characteristics	Presence	Agenda-Setting
1. Q&A mode	Questions and answers, particularly GPs asking patient to concretize their symptoms during the initial phase, and during physical examinations but also in relation to medical treatment	All 10 consultations	GP-led
2. Lecture mode	Communication of information, most pronounced when GPs elucidate results of technological tests and physical examinations, explain etiological issues, and present treatment options	All 10 consultations	GP-led
3. Probabilistic mode	Medical uncertainty expressed through probabilistic talk, mainly in relation to etiology, treatment options, and prognosis	All 10 consultations	GP-led
4. Competition mode	Patients try to set the agenda or express their views, but meet competition, appears unsystematically for all consultation aspects	Half of the consultations	Mixed
5. Narrative mode	Patients describe problems and their implications for life at work and home, and in relation to treatment options, in long stretches	Two consultations	Patient-led

*Note.* GP = general practitioner.

### Agenda-Setting

In all consultations, the three GP-led modes of interaction (1–3, Table 7) are dominating. These dialogues are characterized by a low level of reciprocity and patient storytelling, and absence of contest. GPs display their upper hand by asking questions and giving information in a lecture-like manner, often combined with indirect expressions of uncertainty through probabilistic talk. Patients do not explicitly express disagreement to the point where doctor’s authority is contested. By accepting a subordinate position, partially or completely, patients recognize doctors’ institutionally based authority position. Their acceptance may take much of its force from their faith in biomedical science. That way, both parties evidence knowledge of and adherence to rules about how “good” and “bad” patients and doctors ought to act. Some patients explicitly state that they act as expected or advised (“I was doing exactly what they told me” and “I’ve learned good exercise”). These findings are consistent with previous research (Pilnick & Dingwall, 2011; Robinson et al., 2016; Stokes et al., 2006).

The relation between modes and agenda-setting seen in our data could easily have been otherwise because it is not questioning and lecturing in itself that makes them GP led, but the ways in which GPs talk. Most importantly, GPs tend to ask closed questions that leave patients with a narrow dialogic space (limited to providing requested information), instead of open questions that invite them to express themselves more freely. GPs also tend to provide information that undermines, or at least not invite, patient engagement, for instance, when they give information about test results through interpretive statements such as “good” or “fine” instead of factual information, which would demonstrate more confidence in patients’ abilities. Within all interactional modes except the narrative one, GPs enforce a medicalized perspective on patients’ problems through a biomedical lexicon. This highlights what is at stake in this arena, and why patient agency is so important.

### Mode Shifts

Throughout each consultation, mode shifts emerge rapidly, usually following specific patterns. Mode shifts toward the dominating modes (1–3, Table 7) map on to the chronology of the consultations, and when discussion topics change, so does the mode. The Q&A mode is typical in opening phases, when GPs explore patient’s symptoms. The lecture mode and the probabilistic mode are most prominent when test results, causal factors, and treatment options are presented and discussed.

Mode shifts toward the least dominating modes (4 and 5, Table 7), however, appear unrelated to specific discussion topics and consultation phases. The competition mode is primarily initiated by patients who try, but fail, to set the agenda, gain more dialogic space, or claim authority: One patient fails in numerous attempts to discuss her stomach problems rather than her chest problems (which the GP wants to discuss), one patient only receives a brief “bizarre” when he describes a medication side effect, and a patient who worries that her back pain is related to her previous malignant melanoma only receives a brief “I don’t think so, no”, without further explanation. Mode shifts toward a narrative mode are also sparked by patients: One patient creates a mode shift by disclosing previous negative experiences of counseling, another by disclosing how she experiences being bullied at work. That patients who step out of the script and rightfully claim their dialogic space sometimes manage to achieve mode shifts toward a narrative patient-led dialogue is interesting because the narrative mode is a patient-centered mode, which is exactly what *clinicians* are encouraged to move toward. This shows that despite their subordinate position in the field, agency from the patient side is both possible and potentially effective.

## Conclusion

In this article, we offer a theoretically founded exploration of 10 naturally occurring primary care consultations. Based on Bourdieu’s field theory, we have depicted the medical system as a social field that has its own distinct logic, constituted by a set of interrelated hierarchical social positions. Interaction within this field is underpinned by a shared set of informal rules of conduct that are culturally fostered through social interaction, and tacitly claimed as expectations. Relating these theoretical assumptions to the orderliness exhibited in our data (all consultations were predominantly GP led and all patients tacitly accepted a subordinate position) indicates that medical encounters continue to be played out between people who know “the immanent rules of the game”, and generally act as expected. By adhering to the rules of the game, including rules of “acceptable speech”, their interaction constitutes a ritualized “ceremonial order” that is similar across all consultations. This implies that doctor–patient interaction, to some extent, is independent of participants’ “individual sophistication” (Freidson, 1961, p. 10) and that structural forces create boundaries for how to act. Empirical substantiation for these claims has been provided in several studies since 1961, both in primary care (Stokes et al., 2006) and hospital settings (Dixon-Woods et al., 2006), so what does our study add apart from exposing “practice as usual”?

First, our observations reveal a mutual relationship between social structure and social action: Actions are nurtured by structural forces that are reenforced through social interaction. In other words, we are dealing with a manmade *negotiated* order that is shaped, maintained, and changed by those who interact in the social field. Each actor can choose to honor, invert, or disregard the rules of the game. The potential for agency is particularly noticeable in relation to mode shifts toward the mode that gives patients most dialogic space: Narrative modes are sparked by patients who step out of the script and rightfully claim expertise, authority, and responsibility. This means that GPs and patients constitute symmetric or asymmetric relations by enacting them together, and that their interaction is culturally constrained, not culturally predetermined. Reducing the persistent asymmetry requires something from both parties in a reciprocal joint venture: Doctors have to acknowledge patients’ experiences and offer them sufficient dialogic space; patients have to claim their space and tell their stories. This is not easily achieved because their expertise is sourced from different kinds of knowledge: Whereas patients know illness as experienced, doctors know the biomedical account of such experiences. The gap between these two different sources of knowledge is a key component in the structural constraints imposed on both groups of actors by the social field in which their interaction is embedded.

Our main contribution, however, relates to our classification of five dominating modes of interaction. The concept of “mode” points to the ways in which patients and GPs collaboratively are *doing* the interaction. Because the “doing” here mainly consists of exchanges of verbal actions, we classify the modes in relation to speech acts exhibited in the dialogues. While exploring the ways in which modes of interactions moved back and forth in various directions, we found that whereas some mode shifts usually mapped on to the chronology of the consultations, it was patients who initiated mode shifts toward more patient-led interactions. Derived from complete naturally occurring medical consultations covering patients with a wide range of symptoms, we hope that our classification of modes demonstrates general applicability in future research.

## Supplemental Material

sj-pdf-1-qhr-10.1177_1049732321993790 – Supplemental material for Modes of Interaction in Naturally Occurring Medical Encounters With General Practitioners: The “One in a Million” Study Click here for additional data file. Supplemental material, sj-pdf-1-qhr-10.1177_1049732321993790 for Modes of Interaction in Naturally Occurring Medical Encounters With General Practitioners: The “One in a Million” Study by Olaug S. Lian, Sarah Nettleton, Åge Wifstad and Christopher Dowrick in Qualitative Health Research
