# Supplementary material for: Modes of Interaction in Naturally Occurring Medical Encounters With General Practitioners: The “One in a Million” Study
Source: Qual Health Res. 2021 Mar 4;31(6):1129–43. doi: 10.1177/1049732321993790 (PMC8114434; doi:10.1177/1049732321993790)
Supplement: sj-pdf-1-qhr-10.1177_1049732321993790 – Supplemental material for Modes of Interaction in Naturally Occurring Medical Encounters With General Practitioners: The “One in a Million” Study [file sj-pdf-1-qhr-10.1177_1049732321993790.pdf]

Supplementary Table 2 Participants

**Patient**			**GP**		**Consultation**		
No.	*Gender*	*Age*	*Gender*	*Age*	*Length*	*Main contact reason(s)*	*Usual GP*
1	Woman	90-99	Woman	30-39	12:05	Neurological	No
2	Woman	70-79	Woman	50-59	09:54	Cardiovascular	Yes
3	Woman	70-79	Woman	30-39	11:38	Digestive	No
4	Woman	50-59	Man	40-49	09:06	Musculoskeletal + general	No
5	Woman	50-59	Woman	40-49	18:28	Psychological	No
6	Woman	40-49	Woman	50-59	15:35	Musculoskeletal	No
7	Woman	30-39	Woman	30-39	07:12	Psychological	Yes
8	Man	30-39	Woman	30-39	18:24	Musculoskeletal + cardio	Yes
9	Man	30-39	Woman	40-49	15:42	Digestive	NA
10	Man	20-29	Man	50-59	09:49	Psychological	Yes
